# Food availability positively affects the survival and somatic maintenance of hibernating garden dormice (*Eliomys quercinus*)

**DOI:** 10.1186/s12983-023-00498-9

**Published:** 2023-05-24

**Authors:** Sylvain Giroud, Marie-Therese Ragger, Amélie Baille, Franz Hoelzl, Steve Smith, Julia Nowack, Thomas Ruf

**Affiliations:** 1 Research Institute of Wildlife Ecology, Department of Interdisciplinary Life Sciences, University of Veterinary Medicine, Savoyenstraße 1, 1160 Vienna, Austria; 2 Konrad Lorenz Institute of Ethology, Department of Interdisciplinary Life Sciences, University of Veterinary Medicine, Vienna, Austria; 3 School of Biological and Environmental Sciences, Liverpool John Moores University, Liverpool, UK

**Keywords:** Costs of torpor, Garden dormice, Temperature, Hibernation, ROS, Telomerase, Climate change

## Abstract

**Background:**

Torpor is an energy saving strategy achieved by substantial reductions of metabolic rate and body temperature that enables animals to survive periods of low resource availability. During hibernation (multiday torpor), the frequency of periodic rewarming—characterised by high levels of oxidative stress—is associated with shortening of telomeres, a marker of somatic maintenance.

**Objectives:**

In this study, we determined the impact of ambient temperature on feeding behaviour and telomere dynamics in hibernating garden dormice (*Eliomys quercinus*) over winter. This obligate hibernator prepares for hibernation by accumulating fat stores but can also feed during hibernation.

**Methodology:**

Food intake, torpor pattern, changes in telomere length, and body mass change were assessed in animals housed at experimentally controlled temperatures of either 14 °C (i.e., a mild winter) or 3 °C (i.e., a cold winter) over 6 months.

**Results:**

When hibernating at 14 °C, dormice experienced 1.7-fold more frequent and 2.4-fold longer inter-bout euthermia, and spent significantly less time torpid, compared to animals hibernating at 3 °C. Higher food intake enabled individuals to compensate for increased energetic costs when hibernating at milder temperatures (14 °C vs. 3 °C), to buffer body mass loss and thus increase winter survival. Interestingly, we observed a significant increase of telomere length over the entire hibernation period, irrespective of temperature treatment.

**Conclusion:**

We conclude that higher temperatures during winter, if associated with sufficient food availability, can have a positive effect on the individual’s energy balance and somatic maintenance. These results suggest that winter food availability might be a crucial determinant for the survival of the garden dormouse in the context of ever-increasing environmental temperatures.

**Supplementary Information:**

The online version contains supplementary material available at 10.1186/s12983-023-00498-9.

## Background

Torpor is an energy saving strategy used by small heterothermic mammals and birds, characterised by a controlled reduction of metabolic rate and body temperature (T_b_), which enables animals to survive energetic bottlenecks [1]. During hibernation, i.e., torpor lasting over several days to weeks, animals can reach a minimum torpid metabolic rate of ~ 4% of basal metabolic rate, in association with a pronounced reduction of their T_b_ [2]. Importantly, hibernation is not a continuous torpid state. Instead, hibernation in most species is structured by successive torpor bouts interspersed by periodic arousals [3–7]. During those arousals, metabolic rate increases drastically, causing T_b_ to return to normothermic levels for a few hours [4, 8] during phases called inter-bout euthermia (IBE). Arousals represent the highest proportion of energy expended during the hibernation process, e.g., 70–80% of expended energy in temperate species [9].

Periodic arousals occur more frequently at higher ambient temperature (T_a_) [10], which increases energy expenditure during hibernation [11, 12]. Most heterotherms rely on body fat stores or food caches over winter that are accumulated prior to hibernation [13–18]. Hence, higher winter temperatures invariably lead to the risk that small hibernators will deplete their fat or food stores before the end of hibernation [19, 20].

Based on models of global climate change, the predicted increase of winter temperatures will act in synergy with other factors and, in the worst case, lead to countless species extinctions [21]. Hibernators are of particular concern, emphasising the need to determine how small hibernators will be affected by warmer winter temperatures. Despite the fact that many seasonal hibernators will likely face negative consequences as a result of the predicted increase in environmental temperature [22], the ability of species to use short bouts of torpor opportunistically seems to be rather beneficial when it comes to surviving temperature extremes and associated increase in food shortages [23].

To date, most studies addressing the impacts of climate change on heterothermy are centred at the level of the whole organism. There is, however, a clear lack of studies investigating cellular or molecular aspects in seasonal hibernators facing events associated with climate change. A reliable measure of somatic maintenance is the change in relative telomere length (RTL). Telomeres are non-coding, repetitive sequences, located at the end of the chromosomes in eukaryotic cells. Telomeres prevent degradation of coding DNA sequences and shorten during mitosis after each cell division [24–29]. During torpor, mitosis and telomere degradation are arrested or drastically reduced [27, 30], whereas at normothermic T_b_, such as during periodic arousals, mitosis is reactivated and telomeres can possibly shorten [30, 31]. Furthermore, periodic arousals, caused by a drastic increase in metabolic rate, are associated with high levels of oxidative stress and substantial increased production of reactive oxygen species (ROS) that can cause accelerated telomere shortening via DNA breaks [4, 32–35]. Telomere length can be restored by active repair mechanisms, e.g., via telomerase [36, 37] or alternative lengthening [38] but these mechanisms are likely energetically costly [33]. Thus, while shortened telomere length is often associated with physiological ageing, studies reporting lengthening of telomeres in relation to improved environmental conditions and lower levels of life-history stress [33, 39–42], suggest that telomeres are rather a biomarker of the current status of individual’s somatic maintenance.

We have recently shown that garden and edible dormice hibernating without food at 14 °C experienced less telomere attrition than individuals hibernating at 3 °C, despite a higher arousal frequency and greater body mass loss [43]. These findings suggest that lower levels of ROS might be produced and/or energy-costly repair mechanisms might be (more) active during hibernation at warmer temperatures. The garden dormouse (*Eliomys quercinus*) sustains its energy requirements during hibernation by relying on its body fat stores, but can also actively forage during the winter [44, 45]; a behaviour that could provide the advantage of buffering telomere attrition and body mass loss during hibernation in winter. To further determine the impact of winter environmental conditions on the somatic maintenance and survival of the individuals, this study built on the previous results and investigated how mild winter T_a_ affects hibernation patterns, telomere dynamics, and energy balance of the garden dormouse when food is available. Thus, torpor patterns, telomere dynamics, body mass changes, and food intakes were recorded in garden dormice hibernating with food, at either 3 °C or 14 °C. Specifically, we hypothesised (1) that garden dormice fed ad libitum are able to partly or fully compensate for telomere attrition during hibernation at low temperatures, i.e., 3 °C, and (2) that *ad-libitum* access to food enables individuals to compensate for higher energy requirements and to limit body mass loss when hibernating at warm temperatures (14 °C),.

## Material and methods

### Animals

We examined 32 (15 females and 17 males) adult garden dormice (2.2 ± 0.2 years old) during hibernation. The animals were born in captivity and raised under natural climatic conditions in outdoor aviaries at the Research Institute of Wildlife Ecology (FIWI) of the University of Veterinary Medicine Vienna, Austria (48° 15′ N, 16° 22′ E). For individual identification all animals were marked at birth with subcutaneous PIT tags (Tierchip Dasmann, Tecklenburg, Germany). In autumn 2017, animals were transferred from the aviaries to the laboratory. The mean body mass of individuals at hibernation onset was 140.0 ± 18.7 g.

### Experimental design

Given their natural duration of hibernation of approximately 6 months [46], experiments were carried out between 21st of September 2017 and 15th of March 2018. Dormice were placed in ventilated cooling units (Liebherr GKv 5730). In each cooling unit, eight animals (with balanced sex ratio) were housed in “holding cages” consisting of a standard laboratory mouse cage (36L x 20T x 14H cm) and a metal grid-lid. Each holding cage was connected to a nest box with a wooden-lid through a connector as previously described in Nowack et al. [43]. Garden dormice were divided into two temperature groups: 16 animals were kept at about 3 °C to mimic a cold winter [mean T_a_: 3.70 ± 0.67 °C (SE: 0.003 °C) and 3.60 ± 0.63 °C (SE: 0.003 °C)] and 16 other animals at 14 °C [mean T_a_: 14.00 ± 1.73 °C (SE: 0.01 °C) and 14.19 ± 1.63 °C (SE: 0.01 °C)] mimicking a mild winter. Experiments were split into four periods (period 1: 21.09.–09.11.2017; period 2: 09.11.–14.12.2017; period 3: 14.12.2017–08.02.2018; period 4: 08.02–15.03.2018). Period 1 represents the beginning-, period 2 and 3 the main- and finally period 4 the end of the hibernation season. The first period lasted 7 weeks (49 days), the second and fourth period lasted 5 weeks (35 days) and the third period was 8 weeks (56 days). Each period began and ended with the recording of body mass and the sampling of buccal swabs (see below), resulting in five sampling points. After the third period, one female of the 3 °C group was excluded from the experiments due to low body mass (70.1 g; despite ad libitum food). This animal was transferred into a warm room kept at approximately 22 °C, receiving ad libitum access to food (see below) and water for the rest of the winter, before returning to the colony. Hibernating animals had also *ad-libitum* access to food and water, and food intake was measured during the whole duration of experiments (see below). At the end of the winter experiments, all animals were returned to the colony.

### Temperature recording and torpor pattern

We used nest temperature as a proxy for T_b_ to estimate torpor use, as described by Willis et al. [47] and used in previous studies in garden dormice [43, 48–50]. In brief, the nest box was equipped with a customized temperature data logger (FIWI, Vienna, Austria; resolution: 0.2 °C, accuracy: ± 0.06 °C), which measured the temperature of the nest box every minute. Nest boxes were big enough for one dormouse to fit completely inside but small enough that it had to sit directly on the thermologger. The bottom of the nest boxes were covered with a thin layer of hay to provide familiar nesting conditions but to still ensure the contact between the animal and data loggers. To mimic normal conditions during hibernation, animals were kept under constant darkness and at a near stable temperature in the cooling units. Torpor bout duration, arousal frequency and IBE duration were computed with a self-written R script [51], using a threshold of 5 °C for the 3 °C group and a threshold of 16 °C for the 14 °C group to compute arousal (> 5 or > 16 °C) and torpor periods (< 5 or < 16 °C). These two thresholds were chosen as a conservative approach to avoid false data due to slight temperature variations.

### Food intake

Individual food intake was measured over the entire duration of the experiment. Each cage was equipped with a bowl of food pellets for rodents (ssniff®HA, ssniff GmbH, Soest, Germany) and a cushion-formed piece of jelly-agar (10 g Agar–Agar in 1 l of water) for water availability. Food pellets were dried for ~ 14 h at 50 °C and weighted before as well as after each period. Pellets and water cushions were exchanged every 1–3 weeks during cage exchange. To prevent animals from disturbance the cooling units were opened under special care, using red light and fresh cages with fresh food and water-cushions were mounted while the hibernating animals inside the nest were left untouched. Under these conditions, animals were undisturbed and continued their hibernation normally (S.G. and M-T.R. Pers. Obs.). To calculate individual food intake (expressed in grams), the amount of food pellets that were left by the animal was subtracted from the amount that was supplied to the animal.

### Telomere length

DNA samples were collected during all 5 sampling points. Cells were collected with gynobrush brushes (Heinz Herenz Medizinalbedarf, Hamburg, Germany) from the inner cheeks of animals that subsequently rewarmed from torpor. The brushes were gently twisted for 1 min inside each cheek. This method is considered minimally-invasive. The brushes were placed in 1 ml BC buffer and stored in the fridge at 4 °C. The DNA extraction in the laboratory was carried out with the DNeasy Blood and Tissue Kit (Qiagen). RTL was measured with the real-time PCR approach [52], adapted for garden dormice.

A 54 bp portion of the IRBP (inter-photoreceptor retinoid-binding) gene-proto-oncogene was used as the non-variable copy number (non-VCN) gene. Primer sequences for the non-VCN gene were 5′-TGG AAG CAG CTC ATG GGC AC-3′(IRBP_Eq_F1) and 5′-GTG GTG GTA TTG GAG GGG CG-3′ (IRBP_Eq_R1), and telomeric primer sequences were 5′-CGG TTT GTT TGG GTT TGG GTT TGG GTT TGG GTT TGG GTT-3′ (tel 1b) and 5′-GGC TTG CCT TAC CCT TAC CCT TAC CCT TAC CCT TAC CCT-3′ (tel 2b) as in Hoelzl et al. [40]. The following procedure as described by Hoelzl et al. [40] was respected. Non-VCN gene and telomere PCRs were carried out in separate runs with 20 ng DNA per reaction, 400 nmol l^−1^ of each primer in a final volume of 20 μl containing 10 μl of Promega Cybergreen GoTaq® qPCR Master Mix (Cat. Nr. A6001/2; Promega, Madison, USA). PCR conditions for IRBP were 10 min at 95 °C followed by 40 cycles of 10 s at 95 °C, 20 s at 63 °C and 20 s at 72 °C. PCR conditions for the telomere primers were 10 min at 95 °C followed by 40 cycles of 10 s at 95 °C, 20 s at 56 °C and 20 s at 72 °C. In each run, a final melting step was included with the temperature ramping from 65 to 95 °C, at 1 °C steps. Two reference standard samples (standard A and standard B) were included in every run and compared with all ratios of telomere to non-VCN gene. A non-template control was included as well in every run. To minimize pipetting errors, reactions were prepared using the Qiagility PCR robot (Qiagen, Germany). Cycling was conducted on a Rotorgene Q quantitative thermocycler (Qiagen, Germany). For analysis of the non-baseline corrected raw qPCR data, the software LinRegPCR (2012.0) was used. RTL was calculated using a modified formula from Ruijter et al. [53], where E is the qPCR efficiency and Ct the cycle threshold. The subscript ST refers to the telomere reaction of the standard sample, SC to the control gene reaction of the standard sample, T to the telomere reaction of the target sample and C to the control gene (IRBP) reaction of the target sample: RTL = (E_T_^CtT^/E_ST_^CtST^)/(E_c_^CtC^/E_SC_^CtSC^).

The mean qPCR efficiency was calculated via the amplification plot method [54] which gives lower but more accurate estimates of efficiency than standard curve based methods [55, 56]. For the non-VCN gene and telomere reactions, mean qPCR efficiencies were 91.0% and 78.6%, respectively.

Reference standard samples were included in every run to provide the relative telomere ratios to the non-VCN gene. Liver tissue was used for standard sample one (S1) (RTL = 1) and standard sample two (S2) which was used to control for run-to-run variability. A negative control was included in every run to control for possible contaminants in the reagents.

The intraclass correlation coefficient (ICC) was calculated as a measure of reliability within and between the runs, as suggested by Koo and Li [57]. ICC estimates and their 95% confident intervals for sample triplicates were calculated in R Version 3.5.1 [51]. Intra-rater ICC was calculated on all included data points based on a single-rating, absolute-agreement, 2-way mixed-effects model (ICC in library ‘irr’, Gamer et al. [58]). Intra-assay ICC for Ct values for telomere assay was 0.99 [*p* < 0.0001, 95% (CI 0.98–0.991)] and for cMYC 0.94 [*p* < 0.0001, 95% (CI 0.92–0.96)] showing an excellent degree of reliability. The ICC for inter-assay was calculated for the standard samples based on a mean rating (k = 3), agreement, 2-way mixed-effects model. Interrater ICC for Ct values for telomere assay was 0.72 [*p* < 0.0001, 95% (CI 0.26–1.0)] and for cMYC 0.99 [*p* < 0.0001, 95% (CI 0.91–1.0)] showing a moderate-to good degree of reliability and excellent degree of reliability respectively. As all samples per individual were run on the same plate, inter-assay variability should have minimal effect on our longitudinal results.

The intra-assay coefficient of variation among replicates (intra-assay variation), an estimate of system precision, was further used to assess reproducibility. Mean intra-assay CV for Ct values of the non-VCN gene and telomere assay were 0.39 and 0.67%, respectively.

Considering the correlation between initial RTL and telomere shortening RTL0 was included, as a correcting factor in all models that were run to identify factors that influenced the change in RTL. Including initial RTL as a covariate in statistical models corrects for the RTL-typical “regression to the mean”, i.e., changes tend to be larger when the initial value is extremely high or low [40].

### Statistical data analyses

Due to a low body mass one animal was excluded from the experiments after period 3. Thus, for the statistical analysis of body mass change, torpor patterns, food intake and RTL change of period 4 the sample size was reduced to 15 individuals in the 3 °C group. Moreover, one sample (RTL0) of one animal and two further samples of another animal (RTL1 and RTL2) of the 14 °C group were excluded due to qPCR reaction failures, reducing the RTL sample size (of all five sampling times) of the 14 °C group to 77. Thus, the total RTL sample size (of both groups) was 156. Data are presented as mean ± standard deviation.

Statistical analyses were conducted using R (Version 3.5.0) [51]. Normal distribution of model residuals and homogeneity of variances were tested using Shapiro–Wilk test, qqPlot and Levene’s test, respectively (Shapiro Wilk test in library ‘stats’; qqPlot and Levene’s test in library ‘car’, Fox and Weisberg [59]. Linear mixed-effects models (lme) were used to test effects of time (time points 1–5) or period (1–4), group (3 °C or 14 °C), as well as the interaction between time and group, or period and group on body mass, total and mean IBE durations, arousal frequency, total and mean torpor durations, and food intake, with animal ID as a random factor. A linear mixed-effects model was also used to test the effect of group, all hibernation parameters (i.e., IBE, arousal frequency, torpor bout duration), body mass, and food intake on the overall RTL change. “Period” was used for data recorded over time (total and mean IBE durations, arousal frequency, total and mean torpor durations and food intake) and “Time” was used for data recorded only at discrete sampling dates (body mass and RTL). Further, we ran a type III sum-of-squared ANOVA followed, when necessary, by a post-hoc Tukey-like all comparisons test (glht in library ‘multcomp’, Hothorn et al. [60]) to determine significances between groups and detailed information on time-course significances within groups. We also conducted regression analyses between food intake and IBE duration by using spearman’s rank correlation (function ‘corr.test’). In all models, “Group” and “Period” were entered as factors. “Time” was used as a factor in linear mixed-effects model on body mass, and as a continuous variable in the linear mixed-effects model on RTL. For statistical analyses of RTL, the linear mixed-effects model included RTL0 (initial RTL) as a covariate to correct for the “regression to the mean” phenomenon [40]. Possible collinearity of variables was tested by using the Variance Inflation Factor (VIF) [61], and when needed, the number of variables was reduced. This was the case for the food intake, which was then excluded from the model, due to high correlation with IBE duration. The following variables were included in the model: “Group”, “RTL0”, “body mass change”, “IBE duration” and “arousal frequency”. We then performed a model selection based on Akaike’s information criterion corrected for small sample size (AICc; Akaike [62]) and an ANOVA type II, with the variables of the best model only (dredge in library ‘MuMIn’, Barton [63]; Anova in library ‘car’). To test the effect of “Time” on RTL, we further applied a linear mixed-effects model (including “RTL0” and a “Group” and “Time” interaction) with a model selection followed by an ANOVA type II on the best model (∆ AIC = 0) only (lme in library ‘nlme’ Pinheiro et al. [64]; dredge function in library ‘MuMIn’ Barton [63]; Anova in library ‘car’). To examine the significance of group difference of initial RTL and the overall RTL change (between RTL0 and RTL4), we applied linear mixed-effects models (lme in library ‘nlme’, Pinheiro et al. [64]) with animal ID as random factor, followed by an ANOVA type III.

## Results

### Body mass loss and food intake

**Fig. 1:**
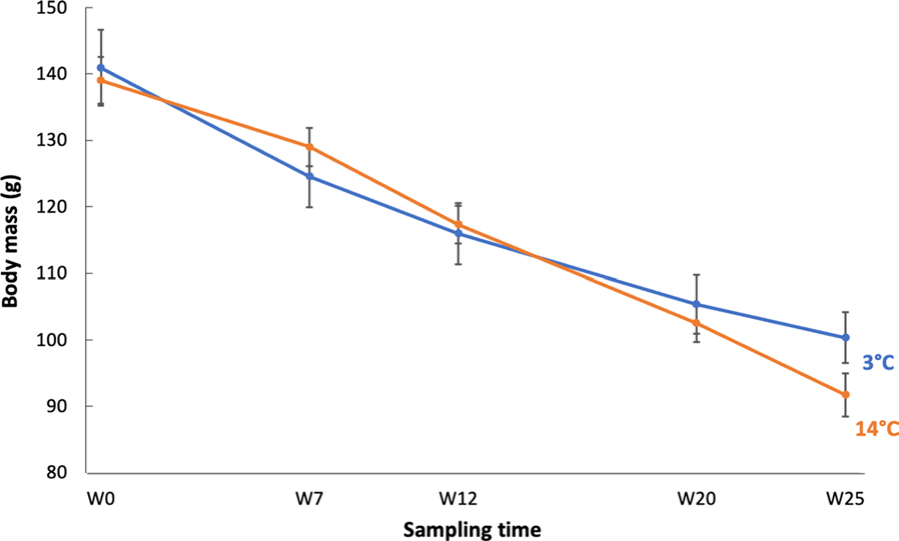
Body mass change of garden dormice hibernating with food at different temperatures during winter. Body mass loss of animals hibernating at 3 °C (blue line) and 14 °C (orange line) over all four periods (weeks). Time was considered as a continuous variable in the model. Post-hoc analyses showed no significant differences between groups at different sampling times: W0 = Week 0, W7 = Week 7, W12 = Week 12, W20 = Week 20, W25 = Week 25

Dormice of both groups had a similar body mass at the beginning of the experiment (3 °C group vs. 14 °C group: 140.9 ± 22.9 g vs. 139.0 ± 14.1 g, t = 0.72519, *df* = 25, *p* = 0.48) and showed a linear loss of body mass over winter (3 °C: − 43.3 ± 8.8 g vs. 14 °C: − 47.3 ± 12.0 g). There was a significant interaction of group and time for body mass loss (Table 1), however, post-hoc tests did not reveal significant differences between groups at any of the timepoints (Fig. 1).

**Table 1:** Effect of temperatures on hibernating patterns, body mass, and food intake of dormice during winter

Response variables	Terms	X^2^	*df*	*p* values
Body mass	Group	0.1	1	0.74
	Time	503.1	4	< *0.001*
	Group:Time	15.2	4	< *0.001*
Total IBE duration	Group	60.7	1	< *0.001*
	Period	9.9	3	*0.02*
	Group:Period	12.8	3	< *0.01*
Mean IBE duration	Group	51.1	1	< *0.001*
	Period	10.1	3	*0.02*
	Group:Period	34.2	3	< *0.001*
Arousal frequency	Group	7.5	1	< *0.01*
	Period	14.0	3	< *0.01*
	Group:Period	28.1	3	< *0.001*
Total TBD (per week)	Group	136.9	1	< *0.001*
	Period	30.9	3	< *0.001*
	Group:Period	52.5	3	< *0.001*
Mean TBD	Group	25.2	1	< *0.001*
	Period	76.5	3	< *0.001*
	Group:Period	18.0	3	< *0.001*
Total FI (per week)	Group	25.0	1	< *0.001*
	Period	35.1	3	< *0.001*
	Group:Period	7.0	3	0.07

Parameters of analyses of variance for the effects of group (3 °C vs. 14 °C), time (weeks) or period (1–4), and their interaction on body mass, total inter-bout euthermia (IBE) duration, mean IBE duration, arousal frequency, total torpor bout duration (TBD), mean TBD, and total food intake (FI) over the hibernation experiments. Significant *p* values are indicated in italic

**Fig. 2:**
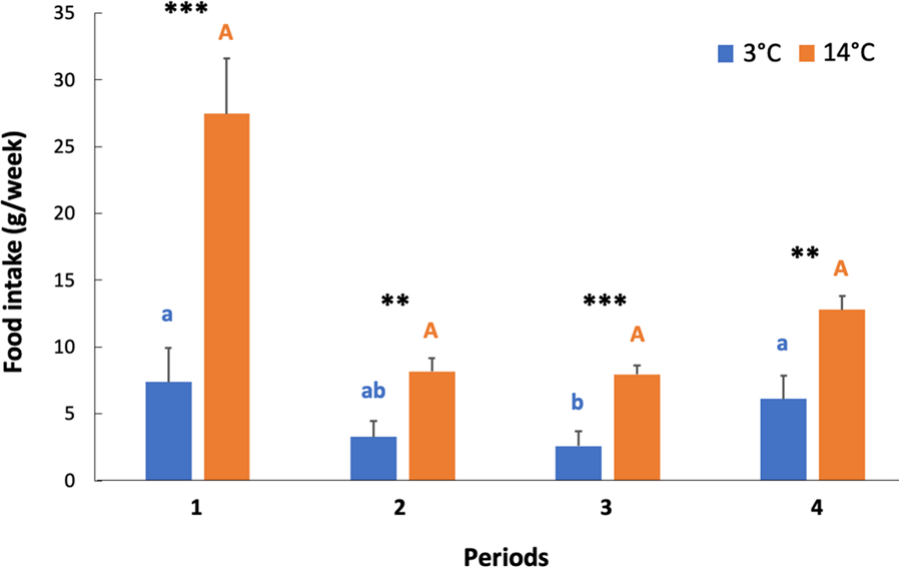
Food intake in hibernating garden dormice according to temperatures during winter. Total food intake (grams per week) for all four periods. Significant differences (*p* < 0.05) are denoted by different superscripts. Period-differences for the 3 °C group and 14 °C group are indicated by lower-case letters and upper-case letters, respectively. Stars indicate group-differences within periods. ***p < 0.001; **p < 0.01

Individuals kept at 14 °C showed a significant higher food intake in all periods than animals hibernating at 3 °C (Fig. 2). For 14 °C animals, no significant period differences in food intake could be detected (Fig. 2). Animals hibernating at 3 °C displayed a significantly lower food intake in period 3, compared to periods 1 and 4 (Fig. 2). Total food intake was 124.4 ± 165.2 g at 3 °C versus 360.8 ± 118.0 g for 14 °C animals.

### Torpor pattern

**Fig. 3:**
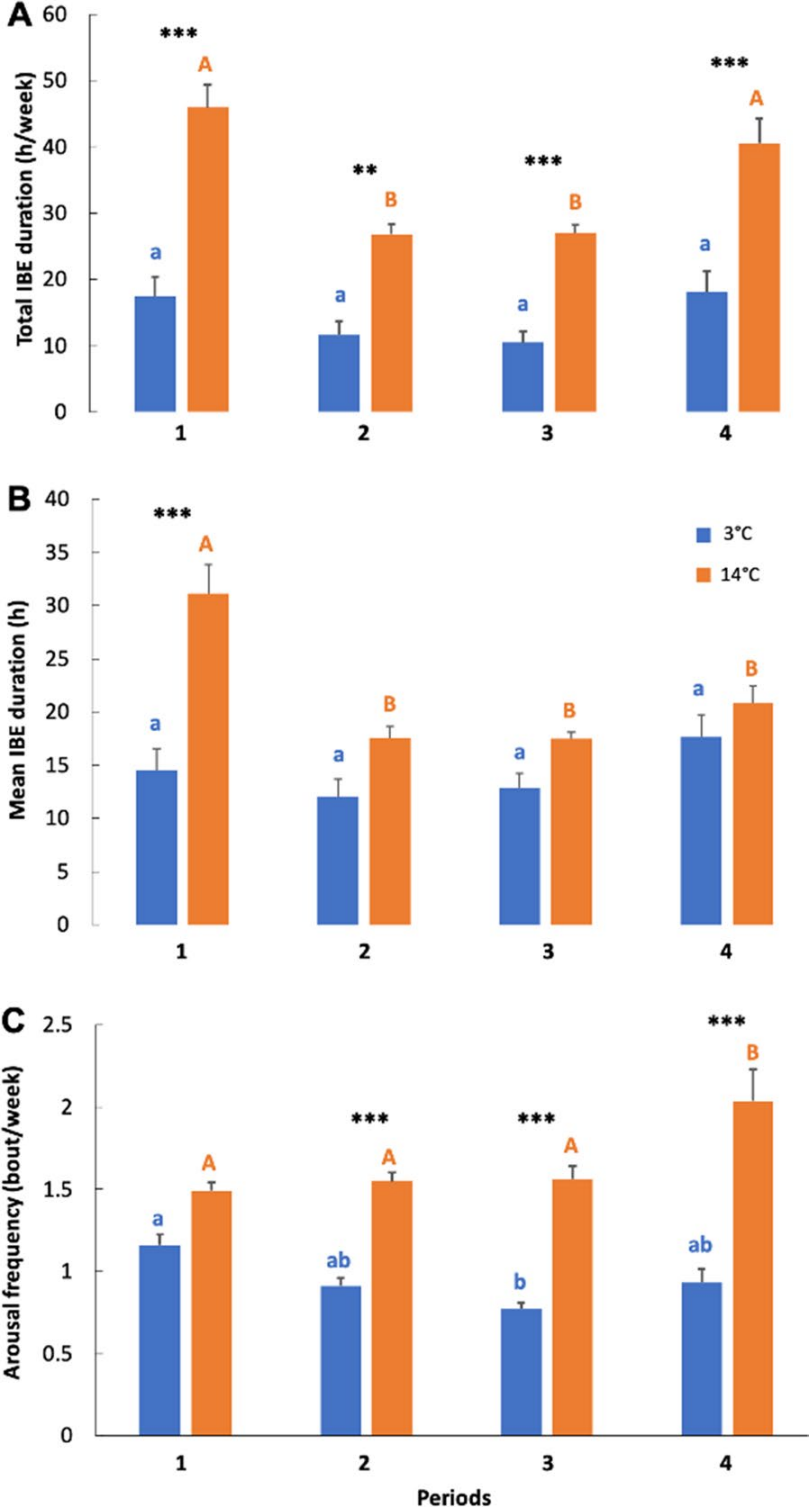
Torpor patterns of garden dormice hibernating with food at different temperatures during winter. **A** Total interbout euthermia (IBE) duration (hours per week), **B** mean IBE duration (h), and **C** arousal frequency (bouts per week) for all four periods. Significant differences (*p* < 0.05) are denoted by different superscripts. Period-differences for the 3 °C group and 14 °C group are indicated by lower-case letters and upper-case letters, respectively. Stars indicate group-differences within periods. ***p < 0.001; **p < 0.01

We found significant differences in total and mean torpor durations between both groups (total torpor duration: 157.3 ± 9.4 days vs. 129.3 ± 10.4 days; mean torpor duration: 7.1 ± 2.2 days vs. 3.4 ± 1.1 days; see Table 2 for values for each period), caused by increased arousal frequency and longer IBE in individuals hibernating at 14 °C. Individuals hibernating at 14 °C showed a 2.4-fold higher total arousal frequency than individuals hibernating at 3 °C and there was a significant group and period interaction for arousal frequency (Table 1; Fig. 3C). While there was no significant group difference at period 1, periods 2, 3, and 4 showed significantly higher arousal frequencies for 14 °C animals versus 3 °C animals (Fig. 3C).

**Table 2:** Torpor variables of garden dormice hibernating with food at different temperatures during winter

Variables	Periods	Temperature groups		*p* values
		3 °C	14 °C	
Total TBD	1	42.7 ± 3.5^a^	27.8 ± 4.3^a^	< *0.001*
	2	32.0 ± 1.5^ab^	29.0 ± 1.2^b^	< *0.001*
	3	51.9 ± 2.1^b^	46.5 ± 1.8^b^	< *0.001*
	4	30.7 ± 2.3^ab^	26.0 ± 3.1^c^	< *0.001*
Mean TBD	1	5.4 ± 1.3^a^	2.8 ± 0.5^a^	< *0.001*
	2	7.2 ± 1.6^b^	4.0 ± 0.7^a^	< *0.001*
	3	8.6 ± 1.7^b^	4.0 ± 1.0^a^	< *0.001*
	4	7.2 ± 2.7^b^	3.1 ± 1.7^a^	< *0.001*

Means and standard deviations of total and mean torpor bout durations (TBD in days) for each temperature group (3 °C and 14 °C) and period (1–4) during the hibernation experiments. *p* values shown in italic correspond to significant differences of total or mean TBD between groups, and different superscripts indicate significant differences of total or mean TBD between periods within a given temperature treatment

**Fig. 4:**
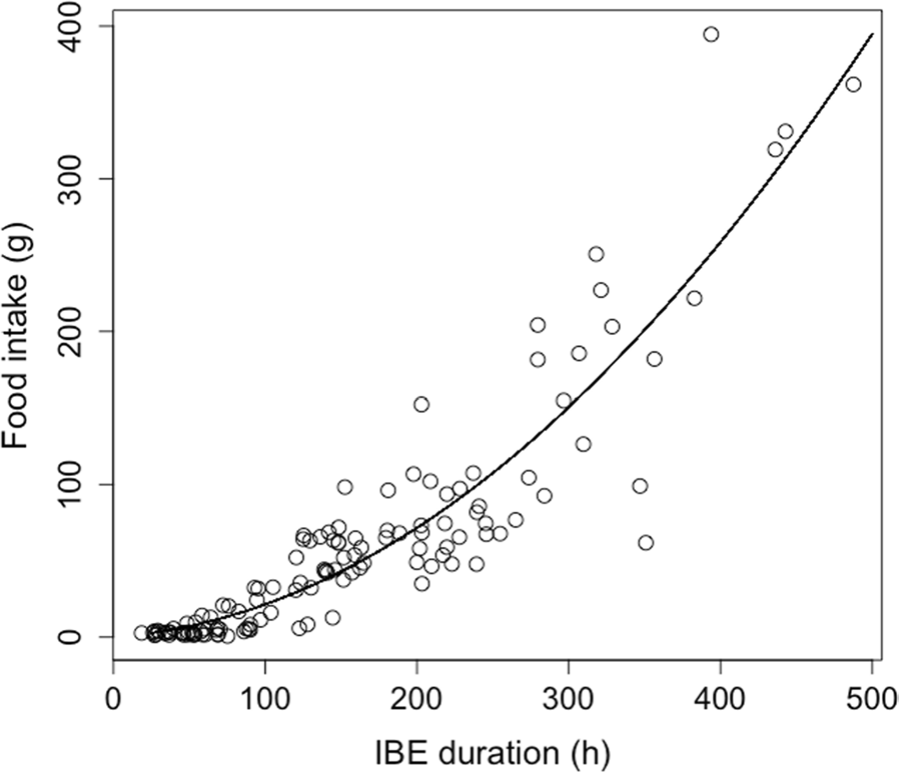
Overall food intake as a function of inter-bout euthermia (IBE) duration. The relationship included individual’s food intakes (grams) and IBE lengths (h) across both temperature treatment groups of garden dormice during the entire hibernation experiments

Mean and total IBE durations were 1.5-fold and 2.4-fold higher, respectively, in individuals hibernating at 14 °C than in the 3 °C group (Mean IBE duration: 21.8 ± 8.6 h vs. 14.2 ± 7.1 h; Total IBE duration: 874.8 ± 138.5 h vs. 360.8 ± 219.8 h). All periods showed a significant group-difference (Table 1; see post-hoc tests on Fig. 3A). Individuals hibernating at 3 °C spent a similar amount of time in IBE in all four periods (Fig. 3A), whereas total IBE duration was highest at periods 1 and 4 for the 14 °C animals (Fig. 3A). Food intake and total IBE duration positively correlated across both groups (S = 29,084, ρ = 0.91, *p* =  < 0.001, Fig. 4).

### Relative telomere length

**Fig. 5:**
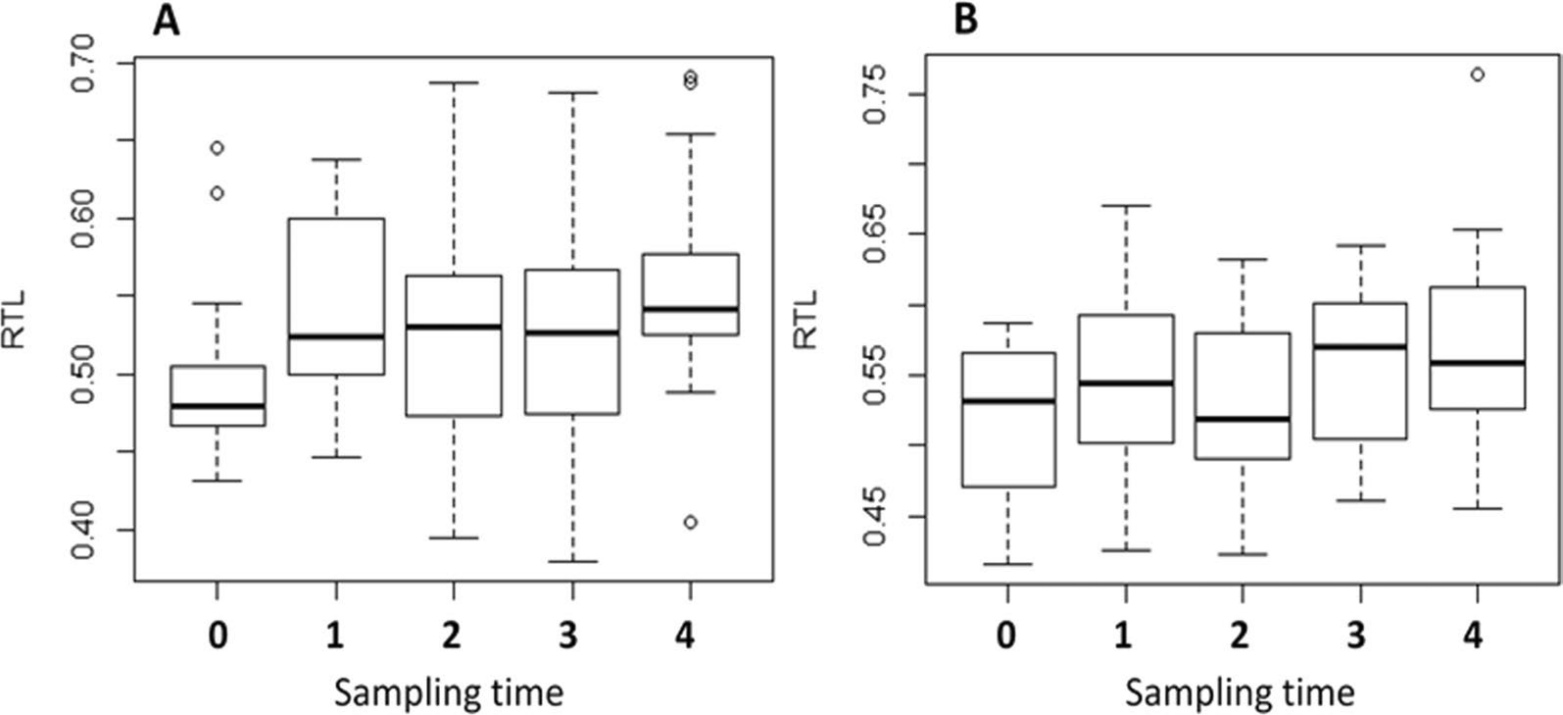
Changes in telomere length of garden dormice hibernating with food at different temperatures during winter. Relative telomere length (RTL) of dormice hibernating at 3 °C (panel A) or 14 °C (panel B) over the five sampling times during the hibernation trial. Sampling time “0” was at the start of the experiments, and times “1” to “4” correspond to samplings performed at the end of each of the four experimental periods

We found a significant increase of RTL over the duration of the study (RTL0-RTL4) (Fig. 5 and Additional file 1: Figure S1; χ^2^ = 11.23, *df* = 1, *p* < 0.001) in individuals of both groups (no group difference: χ^2^ = 3.09, *df* = 1, *p* = 0.08). Total RTL over all four periods during hibernation was best explained by the model including initial RTL and time (Table 3), with a significant effect of both variables on RTL (initial RTL: χ^2^ = 33.5, *p* < 0.001; Time: χ^2^ = 15.9, *p* < 0.001). Although initial RTL was the only variable significantly affecting RTL change (Table 4), the factor that best explained RTL change (besides initial RTL) was arousal frequency (Table 3).

**Table 3:** Best candidate models explaining relative telomere (RTL) or RTL change over the four periods during hibernation in garden dormice

	Model	AICc	∆AIC	Weight
RTL~	RTL0 + time	− 196.91	0.00	0.64
	RTL0 + group + time	− 195.12	1.79	0.26
	RTL0 + group * time	− 193.11	3.80	0.10
RTL change~	RTL0	− 63.07	0.00	0.43
	RTL0 + arousal frequency	− 61.54	1.52	0.20
	RTL0 + group	− 60.93	2.14	0.15
	RTL0 + total IBE duration	− 60.61	2.46	0.12
	RTL0 + body mass change	− 60.21	2.85	0.10

All models were corrected for initial RTL (RTL0). Explanatory variables were time and group for the model on RTL, and were arousal frequency, group, total inter-bout euthermia (IBE) duration and body mass change for the model on RTL change

**Table 4:** Effect of hibernation and temperature on telomere change of dormice during winter

Terms	X^2^	*df*	*p* values
RTL0	13.5	1	< *0.001*
group	0.1	1	0.83
Body mass change	2.1	1	0.15
Arousal frequency	2.9	1	0.09
Total IBE duration	1.4	1	0.23

Parameters of analyses of variance for the effects of initial relative telomere length (RTL0), group (3 °C vs. 14 °C), body mass change, arousal frequency, and total inter-bout euthermia (IBE) duration on RTL change during the hibernation experiments. Significant *p* values are indicated in italic

## Discussion

Our study revealed that while individuals hibernating at warmer temperatures had higher arousal rates and spend more time active, they were able to compensate the high energy requirement via increased food intake, as shown by similar body mass loss in both groups. Furthermore, the presence of food during hibernation allowed dormice hibernating at 3 °C and 14 °C to compensate any shortening of telomere length via repair mechanisms, leading to an increase in telomere length during winter. Such maintenance of somatic integrity during winter hibernation would allow individuals to optimize the successive breeding season as available energy can be fully used to maximize body condition at emergence from hibernation and to ensure a successful breeding.

### The role of hibernation pattern, food intake, and T_a_ on telomere length

In contrast to previous work reporting significant RTL attrition over the course of hibernation, notably in relation to the number of periodic arousals and the duration of IBE [33, 49, 65], this study did not find a significant relationship between telomere dynamics, arousal frequency and IBE duration. Moreover, telomere length after hibernation did not differ significantly between animals that hibernated at mild or cold winter temperatures. While our previous study had shown that dormice hibernating at 3 °C without food shortened telomere [43], dormice hibernating at 3 °C with food in this study steadily elongated their telomeres toward the end of the hibernation season. This suggests that telomere elongation is costly, as previously underlined by Hoelzl et al. [33], and therefore high energy availability either in the form of stored fat or via direct food intake can counteract the negative effects of periodic arousals on telomeres (i.e., on somatic maintenance) during hibernation by allocating metabolic resources to somatic maintenance (telomeres elongation).

In our study, arousal frequency, temperature, total IBE duration, and body mass change were included in the best models explaining RTL variations between individuals, although initial RTL was the only significant predictor variable. Still, it is important to mention that more factors must be considered, with which RTL can be stabilised or elongated. For instance, a fluctuating T_a_ as potentially experienced in a more natural setting may have minimized the cost of arousals and thus the production of ROS and its negative effect on RTL [49].

Alternatively, the availability of food could also have allowed individuals to upregulate their antioxidant defences, leading to dampened concentrations of ROS and to lower oxidative stress level associated with periodic arousals [4, 32], reducing telomere attrition during hibernation. Nowack et al. [43] suggested that the greater extent of telomere attrition in 3 °C animals hibernating without food could be explained by the fact that animals exposed to cold temperatures (3 °C) had to rewarm from deeper torpor than individuals hibernating at mild temperatures (14 °C) resulting in a greater level of oxidative stress. In the present study, dormice from both temperature treatment groups not only prevented telomere attrition during hibernation but also elongated them toward the end of the winter. While telomere elongation was also reported to occur in edible dormice when supplemented with food during the summer [33], the existence of a circannual program aiming at reaching a certain telomere length at emergence from hibernation remains unknown. These findings emphasize that the topic of RTL restoration or elongation is still very complex and that further studies are warranted.

### Dormice hibernating at mild temperatures with food spend less time in torpor

In this study, dormice hibernating at mild temperatures (14 °C) spent overall more time in IBE than individuals hibernating at cold temperatures (3 °C) and our data further revealed a positive correlation between food intake and the time animals spent in IBE. This may reflect the necessity for the 14 °C animals to compensate for higher energy expenditure as warmer T_a_ during hibernation is associated with higher energetic costs, due to T_a_ limiting further reduction of T_b_ and metabolism and leading to an increased number of periodic arousals during winter hibernation [2, 66]. Alternatively, the presence of food during winter might have caused animals to be more active (and less torpid) during hibernation. Individuals hibernating with food in the present study had on average 1.7-fold longer IBE compared to garden dormice hibernating without food at the same T_a_ of 14 °C during winter in our previous study (see Table 1 in [43]). This suggests that food availability reduces the propensity of animals to hibernate, spending less time in torpor and longer time in IBE, underlying the avoidance of torpor use when possible [16, 67, 68]. However, this effect might also have been linked to differences in body energy (fat) reserves, between dormice of the present study and individuals from Nowack et al. [43] that were lighter with ~ 20% lower body mass during winter than individuals from the present study. Previous studies have found that dormice in better body condition, i.e., with more body energy reserves reduce the time spent in torpor (e.g., [68]). Hence, the exact contributions from external food supply and internal energy (fat) reserves to the overall energetics of hibernation in garden dormice still warrant further investigations.

### Effects of warmer winter temperatures on the survival of the garden dormouse

Global mean surface temperature has increased by approximately 0.8 °C over the last century and is likely to continue to increase by 0.3–4.8 °C, over the twenty-first century [22]. Hibernating species are particularly vulnerable to such critical rise in T_a_s [69, 70]. Hence, it is crucial to investigate how hibernators, such as the garden dormouse, react to increasing T_a_, conferring some phenotypic flexibility for individuals to survive the winter, especially when temperatures during the hibernation period are increasingly mild. Given the garden dormouse’s status as near-threatened [71], and the fact that this species experienced the largest decline among all European rodents over the last 30 years [72], it is of high relevance to determine to which extent the flexibility of hibernation phenotypes would enable this species to survive warmer winters. Our results suggest that torpor pattern of garden dormice can be highly flexible and that garden dormice are able to survive uprising mild T_a_ during hibernation—as long as food is available. However, Hallmann et al. [73] reports an alarming 75% decline of flying insect biomass over the last 27 years across several natural reserves in Germany. Although the exact causes for the decline of garden dormice still remains unknown the loss of insect biomass could strongly influence animal population that mainly feed on insects, such as the garden dormouse, enhancing species extinction.

Despite the many other advantageous roles of torpor, such as predation avoidance [23, 74, 75], *Eliomys quercinus* would rather employ less torpor or might even skip hibernation when environmental conditions are good, i.e., high T_a_ possibly accompanied by high food availability [76]. Additionally, mild T_a_ can also lead to ecological advantages including extra-time for the breeding season [77] notably the occurrence of litters during winter with mild temperatures [76]. Although there are already many studies examining the impacts of climate change, there is still a need to improve knowledge concerning animal responsiveness to warmer winter, especially at the physiological and ecological level of this hibernating dormouse species. Hence, it will be of high importance to investigate the phenotypic flexibility of garden dormice in the context of ever-increasing climate and global changes.

## Conclusion

This study highlights that (1) telomeres can be restored during hibernation and (2) individuals can compensate for the high energy requirement when hibernating at mild temperatures, if food is available during winter. Although dormice from both temperature groups were losing body mass, all individuals were able to survive the entire winter. Surprisingly, telomeres increased during the process of hibernation irrespective of the temperature treatment. Animals hibernating at 3 °C allocated the extra-energy -provided via food intake- to their somatic maintenance, whereas animals kept at 14 °C primarily allocated available energy toward regulating their energy balance to survive the winter, along with directing a part of it to their somatic maintenance. To conclude, this data indicates that temperature and food availability during winter have an important impact on hibernating patterns and cellular maintenance.

## Supplementary Information

**Additional file 1: Figure S1**. Change of relative telomere length (RTL) between groups during the entire hibernation experiments. N3°C = 15; N14°C = 15, time = 25 weeks.

## Data Availability

The data underlying this article will be shared on reasonable request to the corresponding author.
