# Supplementary figures and images for: Food availability positively affects the survival and somatic maintenance of hibernating garden dormice (Eliomys quercinus)
Source: Front Zool. 2023 May 24;20:19. doi: 10.1186/s12983-023-00498-9 (PMC10207780; doi:10.1186/s12983-023-00498-9)

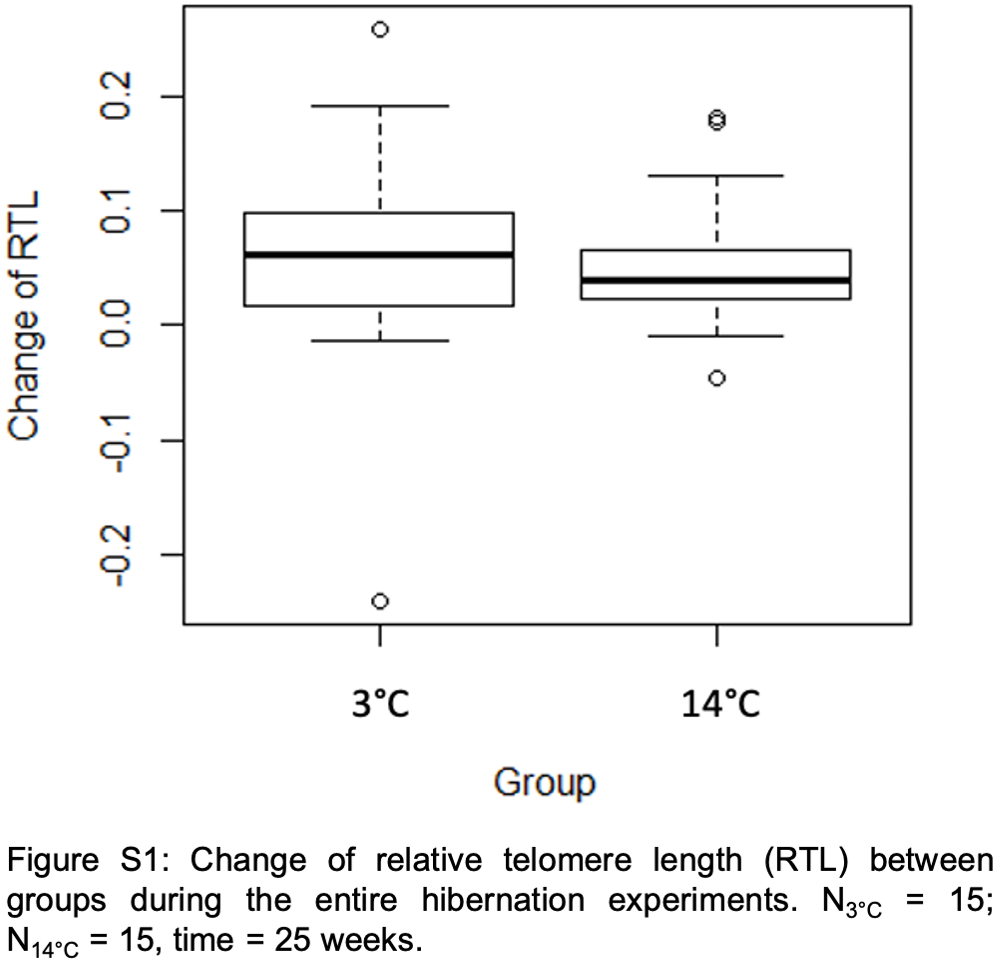

Supplement: Supplementary file 1 — Additional file 1: Figure S1. Change of relative telomere length (RTL) between groups during the entire hibernation experiments. N3°C = 15; N14°C = 15, time = 25 weeks. [file 12983_2023_498_MOESM1_ESM.png]
